# Vaccination Coverage Among Preschool Children in Germany: Trends, Regional Disparities, and Determinants from School Enrolment Examinations in Two Metropolitan Regions

**DOI:** 10.3390/vaccines14070618

**Published:** 2026-07-14

**Authors:** Christopher Michael Dyer, Judith Welker, Kholoud Assaad, Nina Knab, Sanjida Rahman Mim, Maria Karathana, Anne Kühn, Sandra Kronmüller, Peter Tinnemann, Rebecca Zöllner

**Affiliations:** 1 Gesundheitsamt des Rhein-Neckar-Kreises, Kurfürsten-Anlage 38-40, 69115 Heidelberg, Germany; 2 Gesundheitsamt Frankfurt am Main, Breite Gasse 28, 60313 Frankfurt am Main, Germany

**Keywords:** vaccination coverage, preschoolers, public health, infectious diseases

## Abstract

**Background**: Vaccinations are highly effective in preventing infectious diseases, which otherwise present a serious challenge to the health of individuals and public health. To identify potential gaps in vaccination coverage, we examined childhood vaccination levels in Frankfurt am Main (FFM) and the Rhein Neckar district (including Heidelberg city, RNHD) prior to, during and following the COVID-19 pandemic. Moreover, we explored various factors that appear to contribute to lower vaccination levels in specific communities in either or both locations. The results are intended to guide strategies for designing and improving vaccination services for specific groups in local health settings. **Methods**: A retrospective analysis was conducted to examine the vaccination rates of children in Frankfurt am Main and the Rhein Neckar district (including Heidelberg city) by using anonymized data from school entry examinations spanning the years 2017 to 2024. Data pre-processing, analysis, and visualization were performed using R (5.4.2). Frequencies and percentages were calculated for sociodemographic and vaccination rates (the completeness of the vaccination schedule recommended by STIKO for children). Multivariate logistic regression was performed to determine factors with a significant impact on vaccination uptake. **Results**: Multinomial logistic regression with ‘measles-only’ (Measles) as the reference category revealed distinct predictor patterns across vaccination levels. In both regions, children who completed preventive medical check-ups had higher odds of completing the schedule of vaccination plus at least one additional vaccine (ScheduledPlus) versus, measles-only vaccination. In RNHD, children from Eastern European language families and low- and medium-social-status backgrounds showed higher odds of completing the scheduled vaccinations without the recently introduced rotavirus vaccination (ScheduledNotRota), versus measles-only vaccination. In FFM, strong interaction effects were observed between medical examination completion and migration background, with children from non-German birthplaces showing dramatically reduced odds of complete vaccination when preventive care was incomplete. **Discussion**: The proportion of children with a complete vaccination status remained stable over the study period in both locations, with no evidence of post-pandemic decline. Nevertheless, this stability masks important heterogeneity in vaccine coverage patterns across sociodemographic groups. Systematic identification and modeling of interactions between nationality, parental employment, and healthcare utilization tell a compelling “double jeopardy” story that the effects of these risk factors are not additive but synergistic. Regular medical check-ups remain an important factor in ensuring high vaccination coverage among children until they start school and underscore the justification for such established preventive care programs. A nuanced understanding, however, is crucial for moving beyond simplistic, one-size-fits-all public health messaging.

## 1. Background

### 1.1. Vaccination as a Public Health Intervention

Infectious diseases account for a substantial proportion of deaths worldwide [1], highlighting the persistent need for effective public health interventions [2]. Vaccination is one of the most effective public health interventions, substantially reducing the incidence, complications, morbidity, and mortality associated with vaccine-preventable infectious diseases [3,4]. However, its effectiveness depends on several critical factors, including public compliance and trust in vaccinations [5], as well as structural barriers such as access, distribution, affordability, and reimbursement [6].

### 1.2. National Vaccination Policy and Monitoring in Germany

In Germany, the Standing Committee on Vaccination (Ständige Impfkommission, STIKO) issues a schedule of national vaccination recommendations as a guideline for necessary or beneficial vaccinations [7]. Distinct from other vaccines on the schedule, measles is the only vaccine that is legally mandatory for school entry. Immunizations are commonly delivered during routine preventive child health check-ups [8] and documented in paper vaccination cards possessed by individual patients. Prior to starting school in Germany, children are legally required to attend a cost-free school entry health examination, called the “Einschulunguntersuchung” (ESU), typically around the age of five or six. Conducted by pediatricians from the local Health Authority (Gesundheitsamt), it evaluates vaccination status along with the child’s physical health, vision, hearing, motor skills, and social–emotional development to determine school readiness.

There is no local or national register for vaccination in Germany. National estimates using health insurance data indicate coverage levels of approximately 80–90% for individual vaccines, though these data lack granularity for subnational disparities [9]. Therefore, fragmented data sources (e.g., school entrance examinations, population surveys, and health insurance claims data) need to be used to estimate coverage [10].

### 1.3. Predictors of Vaccination Coverage

Despite its effectiveness in preventing infectious diseases, vaccination coverage is influenced by a range of sociodemographic and structural factors [11,12,13,14]. Parent-level barriers—including knowledge deficits, negative attitudes, and practical access constraints—have been identified as key determinants of under-vaccination in high-income settings [15]. Previous studies have identified key predictors of vaccine uptake, including nationality [16,17,18], parental employment status [19], socioeconomic status and the child’s place of residence [20,21]. Also, those who do not fully participate in preventive check-ups are less likely to be fully vaccinated [22]. Understanding these factors is essential for identifying gaps in vaccine coverage and informing targeted public health strategies.

### 1.4. Objectives of the Present Study

This study examined vaccination rates in pre-school children, evaluating the completeness and trends in vaccination coverage among children in accordance with STIKO recommendations from 2017 to 2024. For this, we investigated vaccination uptake for both mandatory and recommended vaccinations, analyzed changes in vaccination rates over time, and compared regional differences between FFM and RNHD. Furthermore, this study aimed to identify factors associated with lower vaccination acceptance and provide insights into the effectiveness of existing vaccination strategies, thereby supporting targeted public health measures and future vaccination policy.

## 2. Methods

### 2.1. Study Design and Data Sources

This cross-sectional study analyzed routine data, collected on the basis of federal state legislation, during school entry health examinations (ESU). The data were collected independently by local public health authorities in two German regions: the Rhein Neckar district (including Heidelberg city, RNHD) in the state of Baden-Württemberg, and the city of Frankfurt am Main (FFM) in the state of Hessen. Pseudonymized datasets were used without individual patients’ identifiers.

These two sites from different metropolitan regions were selected and analyzed separately. This approach provides a broader, complementary picture of vaccination uptake while maintaining similar demographic diversity, particularly regarding nationalities. This reduces confounding effects when examining vaccine uptake within each location. Differences in the availability of socioeconomic data between sites also allowed an assessment of how contextual factors and data depth have the potential to influence results.

In both regions, attendance at the ESU is mandatory for all children prior to school entry. The examinations in FFM are typically performed within the year of school enrollment, while in RNHD, the examinations are typically performed within two years prior to school enrollment. The study data include the available data collected in FFM from 2018 to 2024, and in RNHD from 2017 to 2024. At both sites, the examinations include a comprehensive health assessment carried out jointly by a pediatrician and other health professionals. During the examination, pediatricians evaluate the child’s medical history, physical development and their record of preventive health check-ups. However, the content and process of the examination, as well as data collection procedures, are defined independently by each federal state. Consequently, the datasets from FFM and RNHD have structural differences and cannot be merged or directly compared; all analyses were therefore conducted separately for each region.

### 2.2. Outcome and Predictor Variables

Vaccination status was categorized in both locations into four outcome indicators based on the completion of the schedule of STIKO-recommended childhood vaccines [23].

**ScheduledPlus**: All scheduled vaccinations plus rotavirus or other non-mandatory vaccinations, including tick-borne encephalitis (FSME), hepatitis A and influenza (only RNHD).

**ScheduledNotRota**: All expected scheduled vaccines, excluding rotavirus, due to its inclusion in the schedule only late within the study period (Supplementary Tables S3 and S4).

**Measles**: Measles (legally required for school entry), but otherwise an incomplete vaccination schedule.

**NotMeasles:** Incomplete vaccination schedule with no measles vaccination.

Childhood vaccination against a specific disease was considered to be complete if the child had received the scheduled number of doses according to STIKO [7] by the year of the ESU. The vaccines assessed included varicella (chickenpox), measles, mumps, rubella, meningococcus, Streptococcus pneumoniae, pertussis (whooping cough), tetanus, diphtheria, polio, Hemophilus influenzae type b (Hib), and hepatitis B virus. Records with missing vaccination data (all) were excluded from the analysis (RNHD: 7.1%; FFM: 3.8%).

In each location, a set of categorical sociodemographic predictors from ESU was assessed for analysis. In the RNHD dataset, the initial candidate predictors included the child’s nationality (Nationality), family language (LanguageFamily), parental employment status (Employment), parental education level (Education), family socioeconomic status (Status), the child’s birthplace (BirthplaceChild), child living with one or two parents (Residence), media consumption (Media), speech therapy needs (Speech_therapy), the duration of kindergarten attendance (Kindergarten), and completion of a series of childhood medical examinations, distinct from the ESU examinations, known as U-tests (MedicalExam). In the FFM dataset, predictors included nationality (“NationalityFather”, “NationalityMother”, and “NationalityChild”), birthplace (“BirthplaceFather”, “BirthplaceMother”, and “BirthplaceChild”), language (family language, “LanguageFamily”; primary carer language, “LanguageCarer”; the child’s proficiency, “LanguageAbility”; and the child’s native language, “LanguageNative”), the child’s number of siblings (“Siblings”), attendance of U-test medical examinations (“MedicalExam”), and the duration of kindergarten attendance (“Kindergarten”).

### 2.3. Data Preparation and Handling of Missing Data

For both datasets, categorical variables were recoded into meaningful groups. Missing values for predictors were not omitted but were explicitly categorized as “Unknown” to retain all cases in the analysis and avoid biased estimates from complete-case analysis. Reference categories for all factors were pre-specified based on clinical relevance or to represent the largest or most stable group (marked with an asterisk in Supplementary **Table S2**).

### 2.4. Statistical Analysis

The association between vaccination status and sociodemographic predictors was examined using multinomial logistic regression with four outcome categories, including Measles as the reference category. This approach was adopted in response to reviewer comments to distinguish between (i) fully vaccinated children (ScheduledPlus and ScheduledNotRota), (ii) partially vaccinated children (Measles only), and (iii) children missing essential vaccinations (NotMeasles). In addition to the main effects outlined in Section 2.2, the final RNHD model included significant interactions between LanguageFamily and Nationality, MedicalExam, BirthplaceChild, and Media. The final FFM model included interactions between MedicalExam and BirthplaceChild and Kindergarten. While the models differed, the strategy was consistent across both regions:

First, collinearity among predictors was assessed using Cramér’s V test, and predictors with high collinearity (V > 0.7) were removed from the model.

Second, a main-effects model was fitted using the multinom() function in R (version 4.5.2 [22]).

Third, to explore effect modification, all possible two-way interactions between the remaining main effects were systematically evaluated. Each interaction was tested by adding it to the main effects model, and its significance was assessed using a likelihood ratio test. All interactions with a *p*-value of <0.01 were initially retained for further analysis.

Fourth, a combined model including nominally significant interactions was fitted. Due to increased model complexity, shared variance, and inflated standard errors, many interactions lost significance in this full model. More parsimonious models were then optimized, retaining only those interactions that remained statistically significant (*p* < 0.05) while decreasing AIC and increasing McFadden’s pseudo-R-squared values within the context of the combined model.

Model coefficients were estimated via maximum likelihood. The results are reported as odds ratios (ORs) with 95% confidence intervals (CIs). Odds ratios (ORs) > 1 indicate higher odds of the specified outcome category compared with the reference category (Measles), while ORs < 1 indicate lower odds. Statistical significance for individual predictors was assessed at α = 0.05. Model performance was compared primarily using the Akaike Information Criterion (AIC) and McFadden’s pseudo-R-squared. Prediction accuracy was assessed through classification tables comparing predicted versus observed outcomes.

## 3. Results

### 3.1. Study Population and Descriptive Statistics

The analysis included 42,619 children from the RNHD region and 36,861 children from the FFM region. Participant vaccination characteristics are listed in Table 1.

The proportion of children to complete at least their full schedule of vaccination (scheduled with or without rotavirus vaccination) remained constant over the study period, from 79.6 to 79.9% in RNHD, and from 72.2% to 71.4% in FFM. A comprehensive overview of vaccination coverage for RNHD (Supplementary Table S3) and FFM (**Table S4**) is provided in the supplementary data.

### 3.2. Model Selection and Goodness of Fit

To address the reviewers’ request to distinguish between partially vaccinated and unvaccinated children, we performed multinomial logistic regression with four outcome categories (Table 1), using Measles as the reference group. This approach allowed a direct comparison between children receiving the absolute minimum legally required level of vaccination (Measles) and those receiving the recommended schedule, as well as optional vaccinations (in both scheduled categories) and children not receiving the minimum vaccination requirement (NotMeasles).

The model selection process favored interaction models over main-effects models in both regions. In RNHD, the final multinomial model included eight of 11 predictor variables (“Nationality”, “LanguageFamily”, “Employment”, “BirthplaceChild”, “MedicalExam”, “Residence”, “Status”, and “Media”), with three variables not included due to missing values (Kindergarten) and lack of significant effect (“Speech_therapy” and “Education”). The final RNHD model had an AIC of 72,506 and McFadden’s R-squared of 6.20%, with the most significant interaction effects between LanguageFamily and Nationality, BirthplaceChild, MedicalExam and Media. In FFM, the final model included eight of 13 predictor variables (“BirthplaceChild”, “NationalityFather”, “LanguageFamily”, “Siblings”, “LanguageCarer”, “LanguageAbility”, “MedicalExam”, and “Kindergarten”), with five variables not included due to no significant effect (“NationalityMother”, “BirthplaceMother”, and “LanguageNative”) and collinearity with other variables (“NationalityChild”, “BirthplaceFather”, and “LanguageNative”; **Table S1**). The final FFM model had an AIC of 75,009 and McFadden’s R-squared of 12.54%, with significant interactions between MedicalExam and Birthplace as well as Kindergarten attendance (Table 2).

### 3.3. Key Findings from RNHD

**Figure 1:**
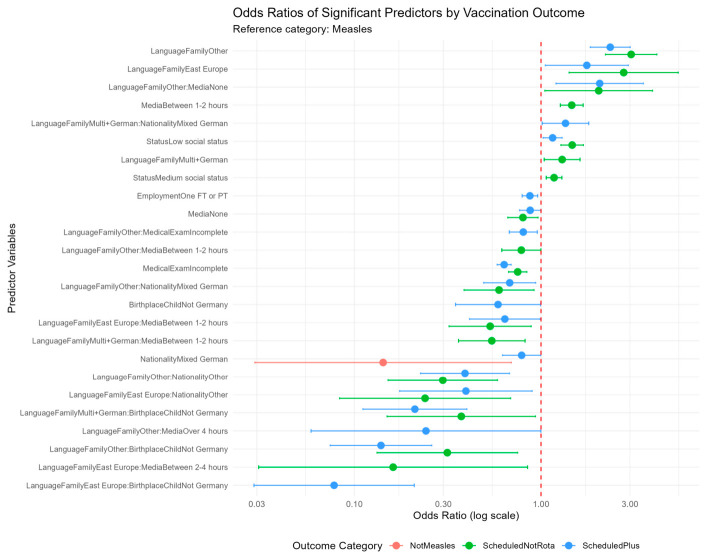
Odds ratios of significant predictors by vaccination outcome category (RNHD). Note: Reference category: Measles. Error bars represent 95% confidence intervals. Red dotted line: OR > 1 indicates higher odds of that outcome versus Measles; OR < 1 indicates lower odds. Plot excludes non-significant effects and unknown predictor values (**Table S5**).

The multinomial logistic regression for RNHD revealed distinct patterns of association across the three vaccination outcome categories relative to the category measles, but otherwise incomplete scheduled vaccinations (**Figure 1** and Supplementary **Table S5**).

**Highest Vaccination Levels (ScheduledPlus vs. Measles)**

Children with Mixed German nationality showed significantly higher odds of completing all scheduled vaccinations plus additional recommended vaccines relative to those with measles only vaccination (OR: 1.35; 95% CI: 1.01–1.80; *p* = 0.039). Complete medical examinations were also strongly associated with scheduled plus vaccination relative to measles-only vaccination (OR: 1.58; 95% CI: 1.45–1.72; *p* < 0.001). Conversely, children from “Other” language backgrounds had 60% lower odds of scheduled plus vaccination compared with measles-only (OR: 0.40; 95% CI: 0.23–0.68; *p* < 0.001), as did those with birthplace unknown (OR: 0.49; 95% CI: 0.15–1.61; *p* = 0.091).

**Scheduled Vaccinations Levels (ScheduledNotRota vs. Measles)**

Several factors were associated with higher odds of completing the established schedule of vaccination versus measles-only vaccination. Children from “Other” language backgrounds had three-fold higher odds of completing the established schedule of vaccinations compared with children with measles-only (OR: 3.04; 95% CI: 2.22–4.18; *p* < 0.001), as did those from Eastern European language families (OR: 2.77; 95% CI: 1.42–5.44; *p* = 0.003). Lower social status was also surprisingly associated with higher odds of scheduled without rotavirus vaccination relative to measles-only vaccination (OR: 1.47; 95% CI: 1.28–1.69; *p* < 0.001).

**No Measles Vaccination (NotMeasles vs. Measles)**

Children without a documented birthplace (“Unknown”) had substantially higher odds of not being vaccinated for measles relative to kids with measles-only vaccination (OR: 6.85; 95% CI: 4.79–9.80; *p* < 0.001). Mixed German nationality, meanwhile, was protective against no measles vaccination (OR: 0.14; 95% CI: 0.03–0.69; *p* = 0.016).

**Interaction Effects**

Significant interaction effects indicated that the effect of family language on vaccination status was modified by nationality, medical examination completion, and media consumption. For example, relative to the odds of receiving measles-only vaccination, the combination of Eastern European nationality with incomplete medical examination showed particularly strong associations with both no measles (OR: 0.52; 95% CI: 0.15–1.81) and scheduled vaccines without rotavirus (OR: 0.27; 95% CI: 0.10–0.73; *p* = 0.010).

### 3.4. Key Findings from FFM

**Figure 2:**
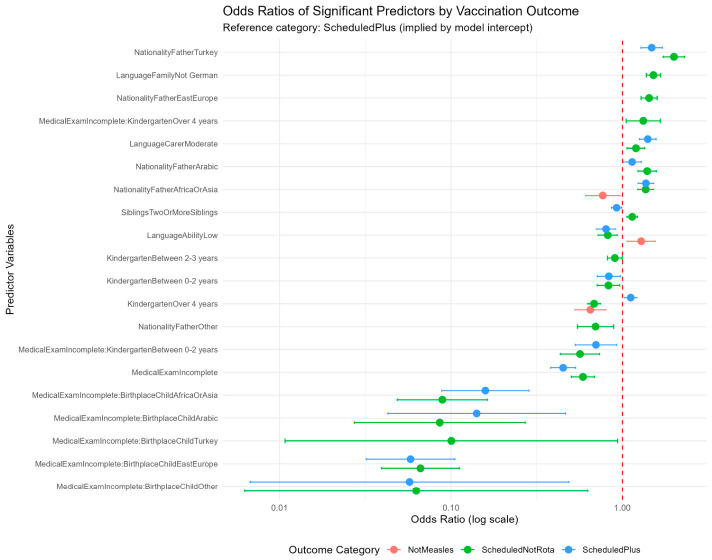
Odds ratios of significant predictors by vaccination outcome category (FFM). Note: Reference category: Measles. Error bars represent 95% confidence intervals. Red dotted line: OR > 1 indicates higher odds of that outcome versus Measles; OR < 1 indicates lower odds. Plot excludes non-significant effects and unknown predictor values.

The multinomial analysis for FFM (N = 35,449) revealed distinct predictor patterns across vaccination categories, with particularly strong interaction effects involving medical examination completion (**Figure 2 and Supplementary Table S6**).

**Highest Vaccination Levels (ScheduledPlus vs. Measles)**

Complete medical examinations in children were strongly associated with the highest level of vaccination coverage compared with children with measles-only vaccination (OR: 2.32; 95% CI: 1.98–2.72; *p* < 0.001). Longer kindergarten attendance (>4 years) was associated with greater odds of the highest coverage of vaccination relative to measles-only vaccination (OR: 1.12; 95% CI: 1.03–1.22; *p* = 0.010). Children whose primary caregiver had a moderate proficiency in the German language had higher odds of vaccination relative to measles-only (OR: 1.41; 95% CI: 1.26–1.57; *p* < 0.001). Paternal nationalities from Africa/Asia (OR: 1.37; 95% CI: 1.23–1.52; *p* < 0.001), Turkey (OR: 1.48; 95% CI: 1.28–1.71; *p* < 0.001), and Arabic states (OR: 1.14; 95% CI: 1.01–1.29; *p* = 0.037) also showed higher odds of vaccination (scheduled plus) relative to measles-only vaccination.

Conversely, lower odds of achieving the highest level of vaccination relative to measles-only vaccination was associated with children: whose caregiver language status was unknown (OR: 0.86; 95% CI: 0.78–0.94; *p* = 0.002); whose own language ability was unknown (OR: 0.76; 95% CI: 0.68–0.84; *p* < 0.001) or low ability (OR: 0.80; 95% CI: 0.70–0.91; *p* < 0.001); and children with two or more siblings (OR: 0.92; 95% CI: 0.86–0.99; *p* = 0.028).

**Scheduled Vaccinations Levels (ScheduledNotRota vs. Measles)**

Non-German family language was associated with higher odds of having completed the expected schedule of vaccination, without rotavirus, relative to measles only vaccination (OR: 1.52; 95% CI: 1.38–1.67; *p* < 0.001). Paternal Turkish nationality showed a particularly strong association with the minimum schedule of vaccinations relative to measles-only vaccination (OR: 2.00; 95% CI: 1.73–2.31; *p* < 0.001), as did paternal nationalities from Eastern Europe (OR: 1.43; 95% CI: 1.29–1.60; *p* < 0.001), Africa/Asia (OR: 1.37; 95% CI: 1.23–1.52; *p* < 0.001), and Arabic states (OR: 1.40; 95% CI: 1.23–1.58; *p* < 0.001). Having two or more siblings (OR: 1.14; 95% CI: 1.06–1.22; *p* < 0.001) and moderate caregiver German ability (OR: 1.20; 95% CI: 1.06–1.35; *p* = 0.003) were also associated with higher odds of completing scheduled vaccinations, without rotavirus, relative to measles-only vaccination.

Unknown caregiver language status showed the strongest positive effect on scheduled vaccination without rotavirus compared with measles-only (OR: 2.71; 95% CI: 2.47–2.98; *p* < 0.001). Unknown or shorter kindergarten attendance was associated with higher odds of the minimum schedule vaccination relative to measles-only: unknown attendance (OR: 0.77; 95% CI: 0.59–0.99; *p* = 0.044), 0–2 years (OR: 0.83; 95% CI: 0.71–0.96; *p* = 0.015), and 2–3 years (OR: 0.90; 95% CI: 0.82–1.00; *p* = 0.045). Incomplete medical examinations were strongly associated with scheduled vaccinations without rotavirus relative to measles-only vaccination (OR: 0.59; 95% CI: 0.50–0.69; *p* < 0.001).

**No Measles Vaccination (NotMeasles vs. Measles)**

Children with an Eastern European birthplace had higher odds of not having measles vaccination relative to measles vaccination (OR: 2.28; 95% CI: 1.00–5.21; *p* = 0.051). Unknown duration of kindergarten attendance was also strongly associated with no measles vaccination relative to only-measles (OR: 2.67; 95% CI: 1.77–4.03; *p* < 0.001), while longer kindergarten attendance (>4 years) was protective (OR: 0.65; 95% CI: 0.53–0.80; *p* < 0.001). Paternal nationalities from Africa/Asia (OR: 0.77; 95% CI: 0.61–0.97; *p* = 0.026) and Turkey (OR: 0.68; 95% CI: 0.46–1.00; *p* = 0.051) showed lower odds of no measles vaccination relative to measles-only vaccination. Unknown caregiver language status, on the other hand, was strongly associated with a child not having the legally mandated measles vaccination relative to measles-only (OR: 2.01; 95% CI: 1.64–2.47; *p* < 0.001), as were children documented with an unknown (OR: 1.49; 95% CI: 1.20–1.85; *p* < 0.001) or low ablity (OR: 1.29; 95% CI: 1.07–1.55; *p* = 0.009).

**Interaction Effects**

The combination of incomplete medical examinations with a non-German birthplace showed dramatically reduced odds of higher vaccination levels. For children with incomplete medical examination, those from Eastern European backgrounds had a 93% lower odds of scheduled plus additional vaccination versus measles-only vaccination (OR: 0.07; 95% CI: 0.04–0.11; *p* < 0.001). Similarly, those from Africa/Asia had 84% lower odds (OR: 0.16: 95% CI: 0.09–0.29; *p* < 0.001), those from Arabic backgrounds had 86% lower odds (OR: 0.14; 95% CI: 0.04–0.47; *p* = 0.001), and those from Other birthplaces had 94% lower odds (OR: 0.06; 95% CI: 0.01–0.49; *p* = 0.009). Similar patterns were observed for scheduled vaccination without rotavirus, with Eastern European (OR: 0.07), African/Asian (OR: 0.09), Arabic (OR: 0.09), and Other (OR: 0.06) birthplaces all showing substantially reduced odds of scheduled vaccination when medical examinations were incomplete relative to measles-only.

Interaction effects were also observed for kindergarten attendance: children with incomplete medical examination who attended kindergarten for 0–2 years had 30% lower odds of having scheduled plus additional vaccination (OR: 0.70; 95% CI: 0.53–0.93; *p* = 0.013) and 43% lower odds of scheduled without rotavirus vaccination (OR: 0.57; 95% CI: 0.43–0.74; *p* < 0.001) relative to the odds of measles-only vaccination. These findings indicate a pronounced “double jeopardy” effect, where migration background combined with healthcare disengagement substantially increases the risk of incomplete vaccination.

## 4. Discussion

This study investigated the vaccination rate of pre-school children and the sociodemographic factors associated with complete childhood vaccination status in two large German districts, Rhein-Neckar-Kreis (RNHD) and Frankfurt am Main (FFM), using data from mandatory pre-school medical examinations. While global trends indicate stagnating or declining childhood vaccination coverage in many high-income countries following the COVID-19 pandemic [24], our findings demonstrate no decline in RNHD and FFM from 2017 to 2024. Nevertheless, our findings, from multinomial logistic regression analysis, also highlight that the level of vaccination coverage is not uniform, but is strongly influenced by a complex interplay of socioeconomic, cultural, and healthcare utilization factors. While at first glance, for example, children with a foreign background often had higher odds of receiving all scheduled vaccines; the positive effect of language and nationality, however, was in many situations significantly modulated by other interacting factors, such as media, birthplace, nationality, kindergarten attendance and medical examinations. The significance of these modeling interactions underscores that the impact of a single risk factor cannot be understood in isolation.

### 4.1. Principal Findings and Comparison with Existing Literature

While global analyses indicate that many high-income countries maintain childhood vaccination coverage above 90% [25], our findings, derived from robust multinomial logistic regression models with Measles as the reference category, reveal distinct pathways to vaccination completion. Rather than a simple vaccinated/unvaccinated dichotomy, children progress through identifiable stages: from no measles vaccination (NotMeasles) to measles-only (Measles), to scheduled vaccinations without rotavirus (ScheduledNotRota), to complete coverage (ScheduledPlus). The predictors of these transitions differ significantly, with complete medical examinations emerging as the strongest consistent predictor of progression to higher vaccination levels in both regions.

In both FFM and RNHD, incomplete completion of preventive medical check-ups (U-tests) was one of the strongest predictors of under-vaccination, with ORs of 1.58 (RNHD) and 2.32 (FFM) for ScheduledPlus versus Measles, and protective effects against NotMeasles. This reinforces that children disengaged from routine pediatric care are at the highest risk, not merely for missing vaccinations, but for lacking the minimum legally required measles protection. Parental employment effects differed by region: in RNHD, single-parent employment reduced odds of ScheduledPlus (OR: 0.87), while in FFM, having two or more siblings reduced odds of ScheduledPlus (OR: 0.92) but increased odds of ScheduledNotRota (OR: 1.14), suggesting household composition effects may operate differently across vaccination stages. This aligns with extensive research demonstrating that socioeconomic deprivation is a major barrier to preventive healthcare, including vaccination, likely due to factors such as reduced health literacy [11,26], limited access to services [5,27], and competing priorities within the family [28]. These check-ups serve not only as an opportunity for vaccination but also as a point of contact where healthcare providers can build trust and educate parents.

The impact of migrant background on vaccination status was also significant, though its manifestation differed between the regions, likely reflecting the different definitions of variables relating to migration background as well as the unique demographic compositions of FFM and RNHD. In both contexts, however, children from non-Western European backgrounds generally faced higher odds of under-vaccination. This finding is consistent with studies across Europe and North America, which have identified migrant status as a risk factor, often linked to language barriers [29,30], differing health beliefs [31], lower accessibility and trust in the healthcare system [5,6,30,32,33,34], and challenges navigating a new country’s medical infrastructure [29,35].

### 4.2. The Critical Role of Multi-Factorial Causes: A Nuanced Understanding

The most novel contribution of our study is the quantification of interaction effects, which reveal that vaccination disparities are synergistic, not additive. In FFM, incomplete medical examinations combined with non-German birthplace produced dramatic reductions in odds of complete vaccination: Eastern European (OR: 0.07; 95% CI: 0.04–0.11), Africa/Asia (OR: 0.16; 95% CI: 0.09–0.29), Arabic (OR: 0.14; 95% CI: 0.04–0.47), and other birthplaces (OR: 0.06; 95% CI: 0.01–0.49) all showed 84–94% reduced odds of ScheduledPlus versus Measles when preventive care was incomplete. For ScheduledNotRota, reductions were 91–94% across all non-German birthplaces. These are not modest interaction effects; they represent the near-elimination of vaccination completion odds when healthcare disengagement coincides with migration background. In RNHD, LanguageFamily × MedicalExam interactions showed similar patterns: Eastern European language families with incomplete medical examinations had 73% lower odds of ScheduledNotRota (OR 0.27). This “double jeopardy” is quantifiable, severe, and demands targeted intervention.

### 4.3. Differences Between Regions and Methodological Strengths

While overarching themes were consistent, the specific interaction structures differed between regions in ways that illuminate local epidemiology. In RNHD, where birthplace data were limited, LanguageFamily emerged as the primary cultural predictor, interacting with nationality, medical examination, and media consumption. In FFM, with richer birthplace data, MedicalExam × Birthplace interactions dominated, suggesting healthcare access barriers are particularly acute for children of foreign-born parents. These differences do not represent contradictory findings but demonstrate how local data availability and demographic composition shape which social determinants are most salient. The FFM model explained more variance (McFadden’s R-squared: 12.6%) than RNHD (6.2%), likely due to predictor richness, though both models identified healthcare engagement as the modifiable core determinant.

### 4.4. Limitations

Our study is subject to several limitations. First, its cross-sectional nature precludes any conclusions about causality. Second, the data were collected for administrative, not research, purposes, meaning some potentially relevant variables (e.g., parental vaccination attitudes and confidence) were unavailable. While validated instruments such as the Vaccination Confidence Scale [36,37] exist for measuring parental attitudes, these data are not routinely captured in school entry examinations. Also, household income and employment structure (e.g., single- vs. dual-earner households) were not available in the administrative dataset and represent important directions for future research, given their established relevance in the broader literature.

The categorization of missing data as ‘Unknown’ retains cases but may obscure the reasons for missing data. Given that in many cases, Unknown values were shown to significantly affect vaccination outcomes, the reasons why data are missing are apparently relevant.

Third, the differences in data collection between FFM and RNHD, while handled by separate analyses, mean that the models are not directly comparable, limiting our ability to draw firm conclusions about regional differences in vaccination determinants.

Finally, residual confounding from unmeasured variables is always possible in observational studies.

### 4.5. Implications for Policy and Practice

The findings underscore the importance of addressing structural and social determinants to improve vaccination uptake and reduce inequities in child health outcomes. Accordingly, the findings have clear implications for public health policy and clinical practice. Interventions must move beyond broad demographic categories and adopt a more targeted approach. Our results strongly support the implementation of integrated programs that simultaneously address socioeconomic needs and healthcare access.

1. **Family language/caregiver and child’s German ability:** Vaccination-specific reminder systems (SMS/app-based; multilingual) tied to the newborn screening or medical examination (U-Tests) schedule, especially relevant for caregivers with lower German proficiency navigating appointment scheduling. This could be paired with interpreter-supported or translated appointment reminder calls from family or children’s doctors when a family is known to have limited German language proficiency or exposure.
2. **Duration of kindergarten attendance:** Kindergarten appears to function as a “catch point” for vaccination status checks. The longer children stay enrolled, the more likely gaps get closed (or over-supplemented). Formalize kindergarten entry and annual re-verification as a vaccination-status checkpoint, not just a one-time entry requirement (as under the Measles Protection Act—Masernschutzgesetz), with mandatory documentation review at each childcare transition, not only at enrollment. This would help catch children who enter with incomplete records and shorten the “0–2 year” window where risk is currently only modestly reduced. For children with a short attendance duration (0–2/3 years), prioritize outreach at the point of childcare registration rather than waiting for later verification cycles, since these children currently show the weakest protective association.
3. **Stage-specific targeted outreach:** Interventions should differentiate by vaccination level. For children lacking measles vaccination, unknown birthplace and caregiver language status predominate, suggesting the need for identity documentation support and interpreter services at first contact. For Measles-only children, kindergarten duration and medical examination completion are leverage points; late kindergarten enrolment should trigger automatic vaccination review. For ScheduledNotRota children, paternal Turkish nationality (OR: 2.00) and non-German family language (OR: 1.52) predominate, indicating the need for culturally specific education around rotavirus and other newer optional vaccines, not generic reminders.
4. **Single-earner households:** Reduce appointment burden for single-earner households by bundling vaccination reminders with existing administrative touchpoints. Vaccination reminders should link to existing touchpoints already used by single-earner families, such as child support administration, childcare enrollment, or U-Test appointments, to reduce the number of separate visits required.
5. **High-risk flag system:** The combination of incomplete medical examination with birthplace in Eastern Europe, Africa/Asia, Arabic-speaking countries, or other regions should trigger automatic coordinated referral for joint medical examination completion plus vaccination review. Current administrative processes (employing separate vaccination and examination tracking) miss this high-risk intersection. Our data suggest this flag system would capture children with 84–94% reduced odds of complete vaccination.

## 5. Conclusions

In conclusion, this study demonstrates that childhood vaccination coverage in Germany is not a binary outcome but exists along a continuum with distinct sociodemographic profiles at each stage. The quantified “double-jeopardy” effects—84–94% reductions in odds of complete vaccination when migration background coincides with healthcare disengagement—demand stage-specific, intersectional interventions. Effective strategies must move beyond one-size-fits-all messaging to (1) flag high-risk birthplace × medical examination combinations for immediate joint intervention; (2) address language barriers for NotMeasles children; (3) leverage kindergarten and U-examination completion for Measles-only children; and (4) provide culturally specific education for ScheduledNotRota families. Future research should test whether coordinated examination–vaccination interventions can break these documented synergistic disadvantage patterns.

## Figures and Tables

**Table 1:** Participant characteristics.

Characteristic	FFM	RNHD
Vaccination category	n (%)	n (%)
ScheduledPlus	12.990 (35.2)	25.361 (59.5)
ScheduledNotRota	12.491 (33.9)	6.235 (14.6)
Measles	8.593 (23.3)	7.083 (16.6)
NotMeasles	1.375 (3.7)	897 (2.1)
Missing	1.412 (3.8)	3.043 (7.1)
Complete records, n (%)	35.449 (96.2)	39.576 (92.9)
Total records, n	36.861	42.619
**Gender**	**n**	**n**
Male	18.986	21.986
Female	17.875	20.633
**Age**	**months**	**months**
Mean (SD)	73.0 (3.48)	61.2 (5.36)
Range (min.–max.)	49–97	49–93
n	36.861	42.619
SE	0.018	0.026

**Note.** For FFM (records 2018–2024); for RNHD (records 2018–2025). Dark gray—table headings; light gray—table section headings. Percentages for vaccination categories were calculated relative to total records. SD—standard deviation; SE—standard error. The variable “Other” represents other countries/nationalities or stateless individuals.

**Table 2:** Final model performance metrics comparing the performance of the main-effects model versus intermediary and final interaction models for RNHD and FFM.

Model (Location)	N	Para- Meters	AIC	Delta_AIC	Pseudo-R2 Mc- Fadden
Final RNHD	39,576	243	72,506	0.0	6.20%
Intermediate 10 RNHD	39,576	243	72,518	12.0	6.18%
Intermediate 5 RNHD	39,576	225	72,545	39.0	6.10%
Main effects RNHD	39,576	81	72,606	100.1	5.65%
Final FFM	35,449	147	75,009	0.0	12.54%
Intermediate 6 FFM	35,449	321	75,111	102.2	12.81%
Intermediate 3 FFM	35,449	381	75,198	188.5	12.85%
Main effects FFM	35,449	87	75,394	385.1	11.95%

Note. AIC = Akaike Information Criterion; Delta(Δ)_AIC = difference in AIC relative to the best-fitting (Final) model within each location. Sample sizes differ between RNHD and FFM models due to listwise deletion of missing cases. The Final model in each location, highlighted in light gray, was retained as the preferred specification for each location on the basis of minimum AIC, consistent with the principle of parsimony; note that for FFM, the Intermediate 3 model achieved a marginally higher pseudo-R^2^ (12.85%), despite a substantially higher AIC (ΔAIC = 188.5), reflecting the cost of its additional parameters (381 vs. 147).

## Data Availability

The data presented in this study are available upon reasonable request from the corresponding author due to ethical and data protection regulations.

## Supplementary Materials

The following supporting information can be downloaded at: https://www.mdpi.com/article/10.3390/vaccines14070618/s1. Table S1: Cramer’s V test for association between variables (0 no assoc. 1 perfect assoc.); Table S2: Vaccination coverage for each variable value; Table S3: Vaccine status among pre-school children in RNHD (2017–2024); Table S4: Vaccine status among pre-school children in FFM (2018–2024); Table S5: Multinomial logistic regression analysis of vaccination category, relative to only measles, by child, family, and administrative predictors, including interaction effects, for RNHD; Table S6: Multinomial logistic regression analysis of vaccination category, relative to only measles, by child, family, and administrative predictors, including interaction effects, for FFM.
