# Supplementary material for: Vaccination Coverage Among Preschool Children in Germany: Trends, Regional Disparities, and Determinants from School Enrolment Examinations in Two Metropolitan Regions
Source: Vaccines (Basel). 2026 Jul 14;14(7):618. doi: 10.3390/vaccines14070618 (PMC13417971; doi:10.3390/vaccines14070618)
Supplement: Supplementary file 1 [file vaccines-14-00618-s001.zip › vaccines-4336070-supplementary.pdf]

Supplemental material

Sociodemographic characteristics and vaccination status of examined preschoolers, presented separately by study sites and year of examination.

Contents

Table S1. Cramer's V test for association / collinearity between variables (0 no assoc. 1 perfect assoc.) 1
Table S2. Vaccination coverage for each variable value ... 2
Table S3. Vaccine status among pre-school children in RNHD, 2017–2024 ... 6
Table S4. Vaccine status among pre-school children in FFM, 2018–2024 ... 9
Table S5. Multinomial logistic regression analysis of vaccination category, relative to only measles, by child, family, and administrative predictors, including interaction effects, for RNHD. ... 11
Table S6. Multinomial logistic regression analysis of vaccination category, relative to only measles, by child, family, and administrative predictors, including interaction effects, for FFM ... 15

Table S1. Cramer's V test for association / collinearity between variables (0 no assoc. 1 perfect assoc.)

	Variable1	Variable2	Cramér's V	ChiSq p-value	Categories V1	Categories V2
RNHD	Education	Employment	0.474	0	4	4
	LanguageFamily	Nationality	0.371	0	5	6
	Nationality	BirthplaceChild	0.292	0	6	3
	LanguageFamily	BirthplaceChild	0.243	0	5	3
FFM	LanguageFamily	**LanguageNative**	0.908	0	3	3
	NationalityFather	**BirthplaceFather**	0.887	0	7	7
	**NationalityChild**	BirthplaceChild	0.598	0	7	7
	LanguageCarer	LanguageAbility	0.494	0	4	3
	LanguageNative	LanguageAbility	0.443	0	3	3
	LanguageFamily	LanguageAbility	0.418	0	3	3
	LanguageFamily	LanguageCarer	0.323	0	3	4
	LanguageNative	LanguageCarer	0.323	0	3	4

Note. Effect-size labels follow Cohen's (1988) benchmarks for Cramér's V, adjusted for df. All chi-square tests were significant at $p < .001$. Values above 0.5 were assessed in isolation for significant effects and one from each pair (**in bold**) removed.

Table S2. Vaccination coverage for each variable value

Variable - RNHD	Value	Total	Scheduled Plus (%)	Sched. not Rota (n, %)	Measles (%)	Not Measles (%)
Nationality	West Europe*	34,322	21231 (61.9%)	5333 (15.5%)	4946 (14.4%)	749 (2.2%)
	East Europe	586	188 (32.1%)	34 (5.8%)	269 (45.9%)	3 (0.5%)
	Mixed German	3,587	2213 (61.7%)	380 (10.6%)	659 (18.4%)	38 (1.1%)
	Middle East	377	208 (55.2%)	44 (11.7%)	99 (26.3%)	2 (0.5%)
	Other	2,587	1068 (41.3%)	318 (12.3%)	765 (29.6%)	66 (2.6%)
	Unknown	1,160	453 (39.1%)	126 (10.9%)	345 (29.7%)	39 (3.4%)
Language Family	West Europe*	27,706	17457 (63%)	4096 (14.8%)	4056 (14.6%)	588 (2.1%)
	East Europe	1,273	480 (37.7%)	186 (14.6%)	396 (31.1%)	34 (2.7%)
	Multi+German	6,057	4010 (66.2%)	629 (10.4%)	1029 (17%)	50 (0.8%)
	Other	6,761	3025 (44.7%)	1195 (17.7%)	1470 (21.7%)	160 (2.4%)
	Unknown	822	389 (47.3%)	129 (15.7%)	132 (16.1%)	65 (7.9%)
Education	Graduated Y12*	24,768	15692 (63.4%)	3398 (13.7%)	3985 (16.1%)	468 (1.9%)
	Graduated Y10	7,039	4336 (61.6%)	1192 (16.9%)	952 (13.5%)	99 (1.4%)
	No parent graduate	2,829	1444 (51%)	549 (19.4%)	458 (16.2%)	63 (2.2%)
	Unknown	7,983	3889 (48.7%)	1096 (13.7%)	1688 (21.1%)	267 (3.3%)
Employment	Both 2FT or 2PT*	6,276	3934 (62.7%)	929 (14.8%)	945 (15.1%)	108 (1.7%)
	Both 1FT and 1PT	18,081	11961 (66.2%)	2538 (14%)	2523 (14%)	288 (1.6%)
	One FT or PT	9,503	5425 (57.1%)	1529 (16.1%)	1654 (17.4%)	206 (2.2%)
	Unknown	8,759	4041 (46.1%)	1239 (14.1%)	1961 (22.4%)	295 (3.4%)
Status	High social status*	21,313	13857 (65%)	2833 (13.3%)	3313 (15.5%)	380 (1.8%)
	Low social status	3,714	1929 (51.9%)	726 (19.5%)	571 (15.4%)	76 (2%)
	Medium social status	8,437	5114 (60.6%)	1391 (16.5%)	1253 (14.9%)	142 (1.7%)
	Unknown	9,155	4461 (48.7%)	1285 (14%)	1946 (21.3%)	299 (3.3%)
Birthplace Child	Germany*	13,353	9818 (73.5%)	1170 (8.8%)	1992 (14.9%)	47 (0.4%)
	Not Germany	998	179 (17.9%)	92 (9.2%)	532 (53.3%)	16 (1.6%)
	Unknown	28,268	15364 (54.4%)	4973 (17.6%)	4559 (16.1%)	834 (3%)

Medical Exam	Completed*	31,889	20625 (64.7%)	4780 (15%)	4660 (14.6%)	621 (1.9%)
	Incomplete	8,659	4545 (52.5%)	1345 (15.5%)	1939 (22.4%)	214 (2.5%)
	Unknown	2,071	191 (9.2%)	110 (5.3%)	484 (23.4%)	62 (3%)
Residence	With both parents*	33,784	21228 (62.8%)	4870 (14.4%)	5277 (15.6%)	593 (1.8%)
	With one parent	3,887	2022 (52%)	671 (17.3%)	685 (17.6%)	98 (2.5%)
	Unknown	4,948	2111 (42.7%)	694 (14%)	1121 (22.7%)	206 (4.2%)
Speech Therapy	Not required	32,550	20254 (62.2%)	4661 (14.3%)	5029 (15.5%)	694 (2.1%)
	Required	8,567	4456 (52%)	1372 (16%)	1711 (20%)	161 (1.9%)
	Unknown	1,502	651 (43.3%)	202 (13.4%)	343 (22.8%)	42 (2.8%)
Media	Between 0-1 hour*	26,319	16646 (63.2%)	3730 (14.2%)	4045 (15.4%)	490 (1.9%)
	None	2,787	1888 (67.7%)	284 (10.2%)	478 (17.2%)	41 (1.5%)
	Between 1-2 hours	5,866	3137 (53.5%)	1133 (19.3%)	966 (16.5%)	105 (1.8%)
	Between 2-4 hours	484	241 (49.8%)	70 (14.5%)	120 (24.8%)	9 (1.9%)
	Over 4 hours	124	44 (35.5%)	21 (16.9%)	25 (20.2%)	7 (5.6%)
	Unknown	7,039	3405 (48.4%)	997 (14.2%)	1449 (20.6%)	245 (3.5%)
Kindergarten	Between 0-1 year*	549	216 (39.3%)	49 (8.9%)	219 (39.9%)	5 (0.9%)
	Between 1-2 years	2,640	1696 (64.2%)	259 (9.8%)	579 (21.9%)	15 (0.6%)
	Between 2-3 years	5,730	4045 (70.6%)	549 (9.6%)	949 (16.6%)	23 (0.4%)
	Over 4 years	6,416	4839 (75.4%)	502 (7.8%)	911 (14.2%)	18 (0.3%)
	Waldorf	37	8 (21.6%)	2 (5.4%)	26 (70.3%)	1 (2.7%)
	Unknown	27,247	14557 (53.4%)	4874 (17.9%)	4399 (16.1%)	835 (3.1%)
Gender	Male	21,986	13174 (59.9%)	3180 (14.5%)	3603 (16.4%)	450 (2%)
	Female	20,633	12187 (59.1%)	3055 (14.8%)	3480 (16.9%)	447 (2.2%)
**Variable - FFM**	**Value**	**Total**	**Scheduled Plus (%)**	**Sched. not Rota (n, %)**	**Measles (%)**	**Not Measles (%)**
Birthplace Child	WestEurope*	31833	12625 (39.7%)	12073 (37.9%)	6262 (19.7%)	873 (2.7%)
	EastEurope	1461	105 (7.2%)	175 (12%)	923 (63.2%)	258 (17.7%)
	Other	237	34 (14.3%)	20 (8.4%)	164 (69.2%)	19 (8%)
	AfricaOrAsia	1383	168 (12.1%)	137 (9.9%)	925 (66.9%)	153 (11.1%)
	Turkey	97	11 (11.3%)	19 (19.6%)	54 (55.7%)	13 (13.4%)

	Arabic	373	35 (9.4%)	36 (9.7%)	247 (66.2%)	55 (14.7%)
	Unknown	65	12 (18.5%)	31 (47.7%)	18 (27.7%)	4 (6.2%)
Nationality Child	WestEurope*	29668	11510 (38.8%)	11347 (38.2%)	5956 (20.1%)	855 (2.9%)
	EastEurope	2656	621 (23.4%)	590 (22.2%)	1163 (43.8%)	282 (10.6%)
	Other	182	31 (17%)	20 (11%)	120 (65.9%)	11 (6%)
	AfricaOrAsia*	2250	655 (29.1%)	428 (19%)	1014 (45.1%)	153 (6.8%)
	Turkey	128	42 (32.8%)	21 (16.4%)	51 (39.8%)	14 (10.9%)
	Arabic	519	116 (22.4%)	74 (14.3%)	272 (52.4%)	57 (11%)
	Unknown	46	15 (32.6%)	11 (23.9%)	17 (37%)	3 (6.5%)
Birthplace Mother	WestEurope*	17217	6864 (39.9%)	5973 (34.7%)	3819 (22.2%)	561 (3.3%)
	EastEurope	6404	1975 (30.8%)	2184 (34.1%)	1842 (28.8%)	403 (6.3%)
	Other	731	291 (39.8%)	208 (28.5%)	202 (27.6%)	30 (4.1%)
	AfricaOrAsia	6513	2391 (36.7%)	2098 (32.2%)	1772 (27.2%)	252 (3.9%)
	Turkey	1498	494 (33%)	755 (50.4%)	223 (14.9%)	26 (1.7%)
	Arabic	2918	917 (31.4%)	1217 (41.7%)	686 (23.5%)	98 (3.4%)
	Unknown	168	58 (34.5%)	56 (33.3%)	49 (29.2%)	5 (3%)
Language Native	German*	16189	6662 (41.2%)	5438 (33.6%)	3573 (22.1%)	516 (3.2%)
	Not German	18204	5990 (32.9%)	6617 (36.3%)	4773 (26.2%)	824 (4.5%)
	Unknown	1056	338 (32%)	436 (41.3%)	247 (23.4%)	35 (3.3%)
Nationality Mother	WestEurope*	17161	6751 (39.3%)	6061 (35.3%)	3774 (22%)	575 (3.4%)
	EastEurope	6460	2022 (31.3%)	2181 (33.8%)	1858 (28.8%)	399 (6.2%)
	Other	676	271 (40.1%)	178 (26.3%)	200 (29.6%)	27 (4%)
	AfricaOrAsia*	6169	2266 (36.7%)	1950 (31.6%)	1711 (27.7%)	242 (3.9%)
	Turkey	1956	708 (36.2%)	905 (46.3%)	313 (16%)	30 (1.5%)
	Arabic	2832	904 (31.9%)	1152 (40.7%)	679 (24%)	97 (3.4%)
	Unknown	195	68 (34.9%)	64 (32.8%)	58 (29.7%)	5 (2.6%)
Birthplace Father	WestEurope*	17299	6967 (40.3%)	5900 (34.1%)	3857 (22.3%)	575 (3.3%)
	EastEurope	5154	1547 (30%)	1754 (34%)	1522 (29.5%)	331 (6.4%)
	Other	650	243 (37.4%)	154 (23.7%)	221 (34%)	32 (4.9%)
	AfricaOrAsia	6049	2152 (35.6%)	1980 (32.7%)	1680 (27.8%)	237 (3.9%)
	Turkey	1890	648 (34.3%)	921 (48.7%)	283 (15%)	38 (2%)

	Arabic	3344	1082 (32.4%)	1374 (41.1%)	771 (23.1%)	117 (3.5%)
	Unknown	1063	351 (33%)	408 (38.4%)	259 (24.4%)	45 (4.2%)
Nationality Father	WestEurope*	17596	6978 (39.7%)	6115 (34.8%)	3909 (22.2%)	594 (3.4%)
	EastEurope	5182	1573 (30.4%)	1746 (33.7%)	1535 (29.6%)	328 (6.3%)
	Other	615	232 (37.7%)	147 (23.9%)	202 (32.8%)	34 (5.5%)
	AfricaOrAsia	5600	1991 (35.6%)	1787 (31.9%)	1600 (28.6%)	222 (4%)
	Turkey	2275	832 (36.6%)	1050 (46.2%)	353 (15.5%)	40 (1.8%)
	Arabic	3096	1026 (33.1%)	1229 (39.7%)	729 (23.5%)	112 (3.6%)
	Unknown	1085	358 (33%)	417 (38.4%)	265 (24.4%)	45 (4.1%)
Language Family	German*	15604	6492 (41.6%)	5169 (33.1%)	3445 (22.1%)	498 (3.2%)
	Not German	18683	6107 (32.7%)	6860 (36.7%)	4876 (26.1%)	840 (4.5%)
	Unknown	1162	391 (33.6%)	462 (39.8%)	272 (23.4%)	37 (3.2%)
Siblings	OnlyChild*	6324	2313 (36.6%)	2055 (32.5%)	1683 (26.6%)	273 (4.3%)
	OneSibling	17137	6646 (38.8%)	5730 (33.4%)	4123 (24.1%)	638 (3.7%)
	TwoOrMoreSiblings	11672	3955 (33.9%)	4565 (39.1%)	2701 (23.1%)	451 (3.9%)
	Unknown	316	76 (24.1%)	141 (44.6%)	86 (27.2%)	13 (4.1%)
Language Carer	Advanced*	10986	5710 (52%)	2500 (22.8%)	2581 (23.5%)	195 (1.8%)
	Moderate	4765	2143 (45%)	1266 (26.6%)	1237 (26%)	119 (2.5%)
	Basic	1383	337 (24.4%)	232 (16.8%)	690 (49.9%)	124 (9%)
	Unknown	18315	4800 (26.2%)	8493 (46.4%)	4085 (22.3%)	937 (5.1%)
Language Ability	Low*	3243	735 (22.7%)	765 (23.6%)	1398 (43.1%)	345 (10.6%)
	High	22015	9268 (42.1%)	7151 (32.5%)	5031 (22.9%)	565 (2.6%)
	Unknown	10191	2987 (29.3%)	4575 (44.9%)	2164 (21.2%)	465 (4.6%)
Medical Exam	Incomplete*	5716	1242 (21.7%)	1609 (28.1%)	2462 (43.1%)	403 (7.1%)
	Complete	27668	11468 (41.4%)	10634 (38.4%)	4931 (17.8%)	635 (2.3%)
	Unknown	2065	280 (13.6%)	248 (12%)	1200 (58.1%)	337 (16.3%)

Note. Values are n (%) within the given category unless otherwise indicated. ScheduledPlus: all scheduled vaccinations plus rotavirus or other non-mandatory vaccination; ScheduledNotRota: all expected scheduled vaccines, excluding Rotavirus due to its inclusion in the schedule only late within the study period; Measles: measles, but otherwise incomplete schedule of recommended vaccines; and NotMeasles: incomplete or no measles vaccination.

Table S3. Vaccine status among pre-school children in RNHD, 2017–2024

		Year of examination							
**Vaccine**	**Status**	**2017**	**2018**	**2019**	**2020***	**2021***	**2022***	**2023**	**2024**
		**n = 5,907**	**n = 6,117**	**n = 6,241**	**n = 4,246**	**n = 2,153**	**n = 4,353**	**n = 6,980**	**n = 6,622**
**Diphtheria**	Complete	5,104 (86.4%)	5,289 (86.5%)	5,770 (92.5%)	3,924 (92.4%)	2,045 (95.0%)	4,082 (93.8%)	6,535 (93.6%)	6,181 (93.3%)
	Incomplete	258 (4.4%)	345 (5.6%)	107 (1.7%)	76 (1.8%)	19 (0.9%)	40 (0.9%)	86 (1.2%)	103 (1.6%)
	Unvaccinated	545 (9.2%)	483 (7.9%)	364 (5.8%)	246 (5.8%)	89 (4.1%)	231 (5.3%)	359 (5.1%)	338 (5.1%)
**Hepatitis B**	Complete	4,759 (80.6%)	4,938 (80.7%)	5,515 (88.4%)	3,799 (89.5%)	1,973 (91.6%)	3,970 (91.2%)	6,324 (90.6%)	6,004 (90.7%)
	Incomplete	407 (6.9%)	459 (7.5%)	125 (2.0%)	75 (1.8%)	19 (0.9%)	44 (1.0%)	101 (1.4%)	103 (1.6%)
	Unvaccinated	741 (12.5%)	720 (11.8%)	601 (9.6%)	372 (8.8%)	161 (7.5%)	339 (7.8%)	555 (8.0%)	515 (7.8%)
**Hib**	Complete	5,028 (85.1%)	5,204 (85.1%)	5,665 (90.8%)	3,863 (91.0%)	2,003 (93.0%)	4,018 (92.3%)	6,413 (91.9%)	6,087 (91.9%)
	Incomplete	245 (4.1%)	316 (5.2%)	109 (1.7%)	60 (1.4%)	18 (0.8%)	37 (0.8%)	97 (1.4%)	90 (1.4%)
	Unvaccinated	634 (10.7%)	597 (9.8%)	467 (7.5%)	323 (7.6%)	132 (6.1%)	298 (6.8%)	470 (6.7%)	445 (6.7%)
**Measles**	Complete	5,022 (85.0%)	5,246 (85.8%)	5,447 (87.3%)	3,763 (88.6%)	2,017 (93.7%)	4,099 (94.2%)	6,611 (94.7%)	6,424 (96.7%)
	Incomplete	264 (4.5%)	295 (4.8%)	326 (5.2%)	166 (3.9%)	58 (2.7%)	57 (1.3%)	110 (1.6%)	0 (0.0%)
	Unvaccinated	621 (10.5%)	576 (9.4%)	468 (7.5%)	317 (7.5%)	78 (3.6%)	197 (4.5%)	259 (3.7%)	220 (3.3%)
**Meningococcal C**	Complete	4,952 (83.8%)	5,186 (84.8%)	5,454 (87.4%)	3,768 (88.7%)	1,920 (89.2%)	3,898 (89.5%)	6,161 (88.3%)	6,424 (89.9%)
	Incomplete	*NA*	*NA*	*NA*	*NA*	*NA*	*NA*	*NA*	*NA*
	Unvaccinated	955 (16.2%)	931 (15.2%)	787 (12.6%)	478 (11.3%)	233 (10.8%)	455 (10.5%)	819 (11.7%)	724 (10.1%)
**Mumps**	Complete	5,019 (85.0%)	5,239 (85.6%)	5,443 (87.2%)	3,759 (88.5%)	2,008 (93.3%)	4,087 (93.9%)	6,574 (94.2%)	6,295 (95.1%)
	Incomplete	261 (4.4%)	293 (4.8%)	326 (5.2%)	161 (3.8%)	53 (2.5%)	55 (1.3%)	107 (1.5%)	68 (1.0%)
	Unvaccinated	627 (10.6%)	585 (9.6%)	472 (7.6%)	326 (7.7%)	92 (4.3%)	211 (4.8%)	299 (4.3%)	259 (3.9%)
**Pertussis**	Complete	5,095 (86.3%)	5,281 (86.3%)	5,737 (91.9%)	3,900 (91.9%)	2,032 (94.4%)	4,067 (93.4%)	6,501 (93.1%)	6,159 (93.0%)

		Year of examination							
**Vaccine**	**Status**	**2017**	**2018**	**2019**	**2020***	**2021***	**2022***	**2023**	**2024**
		**n = 5,907**	**n = 6,117**	**n = 6,241**	**n = 4,246**	**n = 2,153**	**n = 4,353**	**n = 6,980**	**n = 6,622**
	Incomplete	243 (4.1%)	320 (5.2%)	105 (1.7%)	67 (1.6%)	14 (0.7%)	36 (0.8%)	82 (1.2%)	93 (1.4%)
	Unvaccinated	569 (9.6%)	516 (8.4%)	399 (6.4%)	279 (6.6%)	107 (5.0%)	250 (5.7%)	397 (5.7%)	370 (5.6%)
**Pneumococcus**	Complete	4,836 (81.9%)	5,078 (83.0%)	5,257 (84.2%)	3,548 (83.6%)	1,736 (80.6%)	3,538 (81.3%)	5,682 (81.4%)	6,424 (89.5%)
	Incomplete	117 (2.0%)	132 (2.2%)	152 (2.4%)	167 (3.9%)	159 (7.4%)	300 (6.9%)	486 (7.0%)	0 (0.0%)
	Unvaccinated	954 (16.2%)	907 (14.8%)	832 (13.3%)	531 (12.5%)	258 (12.0%)	515 (11.8%)	812 (11.6%)	751 (10.5%)
**Polio**	Complete	5,059 (85.6%)	5,259 (86.0%)	5,739 (92.0%)	3,903 (91.9%)	2,025 (94.1%)	4,061 (93.3%)	6,497 (93.1%)	6,154 (92.9%)
	Incomplete	275 (4.7%)	353 (5.8%)	107 (1.7%)	71 (1.7%)	17 (0.8%)	34 (0.8%)	83 (1.2%)	95 (1.4%)
	Unvaccinated	573 (9.7%)	505 (8.3%)	395 (6.3%)	272 (6.4%)	111 (5.2%)	258 (5.9%)	400 (5.7%)	373 (5.6%)
**Rubella**	Complete	5,019 (85.0%)	5,239 (85.6%)	5,443 (87.2%)	3,759 (88.5%)	2,010 (93.4%)	4,090 (94.0%)	6,584 (94.3%)	6,305 (95.2%)
	Incomplete	261 (4.4%)	293 (4.8%)	326 (5.2%)	161 (3.8%)	57 (2.6%)	57 (1.3%)	109 (1.6%)	71 (1.1%)
	Unvaccinated	627 (10.6%)	585 (9.6%)	472 (7.6%)	326 (7.7%)	86 (4.0%)	206 (4.7%)	287 (4.1%)	246 (3.7%)
**Tetanus**	Complete	5,109 (86.5%)	5,295 (86.6%)	5,788 (92.7%)	3,942 (92.8%)	2,052 (95.3%)	4,087 (93.9%)	6,538 (93.7%)	6,180 (93.3%)
	Incomplete	290 (4.9%)	361 (5.9%)	113 (1.8%)	70 (1.6%)	18 (0.8%)	35 (0.8%)	86 (1.2%)	102 (1.5%)
	Unvaccinated	508 (8.6%)	461 (7.5%)	340 (5.4%)	234 (5.5%)	83 (3.9%)	231 (5.3%)	356 (5.1%)	340 (5.1%)
**TBE**	Complete	1815 (30.7%)	1890 (30.9%)	2087 (33.4%)	1518 (35.8)	969 (45.0%)	2040 (46.9%)	3165 (45.3)	2909 (43.9%)
	Incomplete	849 (14.4%)	977 (16.0%)	1064 (17.1%)	745 (17.6%)	321 (14.9%)	628 (14.4%)	986 (14.1%)	1055 (15.9%)
	Unvaccinated	3243 (54.9%)	3250 (53.1%))	3090 (49.5%)	1983 (46.7%)	863 (40.1%)	1685 (38.7%)	2829 (40.5%)	2658 (40.1%)
**Influenza**	Complete	240 (4.1%)	227 (3.7%)	297 (4.8%)	227 (5.4%)	129 (6.0%)	367 (8.4%)	637 (9.1%)	535 (8.1%)
	Incomplete	NA	NA	NA	NA	NA	NA	NA	NA
	Unvaccinated	5667 (95.9%)	5890 (96.3%)	5944 (95.3%)	4019 (94.7%)	2024 (94.0%)	3986 (91.6%)	6343 (90.9%)	6087 (91.9%)
	Complete	132 (2.2%)	133(2.2%)	135 (2.2%)	83 (2.0%)	42 (2.0%)	88 (2.0%)	159 (2.3%)	154 (2.3%)

		Year of examination							
**Vaccine**	**Status**	**2017**	**2018**	**2019**	**2020***	**2021***	**2022***	**2023**	**2024**
		**n = 5,907**	**n = 6,117**	**n = 6,241**	**n = 4,246**	**n = 2,153**	**n = 4,353**	**n = 6,980**	**n = 6,622**
**HAV**	Incomplete	44 (0.7%)	42 (0.7%)	57 (0.9%)	39 (0.9%)	22 (1.0%)	37 (0.9%)	58 (0.8%)	51 (0.8%)
	Unvaccinated	5731 (97.0%)	5942 (97.1%)	6049 (96.9%)	4124 (97.1%)	2089 (97.0%)	4228 (97.1%)	6763 (96.9)	6417 (96.9%)
**Rotavirus**	Complete	1931 (32.7%)	2671 (43.7%)	3508 (56.2%)	2474 (58.3%)	1299 (60.3%)	2628 (60.4%)	4670 (66.9%)	4436 (67.0%)
	Incomplete	47 (0.8%)	73 (1.2%)	140 (2.2%)	122 (2.9%)	56 (2.6%)	100 (2.3%)	171 (2.5%)	216 (3.3%)
	Unvaccinated	3929 (66.5%)	3373 (55.1%)	2593 (41.6%)	1650 (38.9%)	798 (37.1%)	1625 (37.3%)	2139 (30.6%)	1970 (29.8%)
**Varicella**	Complete	4,715 (79.8%)	4,924 (80.5%)	5,179 (83.0%)	3,592 (84.6%)	1,915 (88.9%)	3,859 (88.7%)	6,195 (88.8%)	6,424 (92.4%)
	Incomplete	190 (3.2%)	215 (3.5%)	237 (3.8%)	146 (3.4%)	50 (2.3%)	78 (1.8%)	172 (2.5%)	0 (0.0%)
	Unvaccinated	1,002 (17.0%)	978 (16.0%)	825 (13.2%)	508 (12.0%)	188 (8.7%)	416 (9.6%)	613 (8.8%)	529 (7.6%)

Note. Values are n (%) within the given examination year and vaccine unless otherwise indicated. "Complete" denotes the full recommended dose series for the corresponding antigen at the time of examination; "incomplete" denotes partial series completion; "unvaccinated" denotes no recorded dose. NA = not applicable, as a partial (incomplete) category is not defined for meningococcal C vaccination in this dataset. Measles, mumps, and rubella are reported as separate antigens rather than as a combined MMR category. Hib = Haemophilus influenzae type b; HAV= hepatitis A; Rota=rotavirus; TBE= tick-borne encephalitis. * Sample sizes for 2020-2022 were reduced relative to other years, reflecting disruptions to routine examinations during the COVID-19 pandemic period. Examinations prior to school entry are typically performed within two years prior to school enrollment**.**

Table S4. Vaccine status among pre-school children in FFM, 2018–2024

		Year of examination						
**Vaccine**	**Status**	**2018**	**2019**	**2020***	**2021***	**2022***	**2023**	**2024**
		**n = 6,669**	**n = 6,868**	**n = 4,827**	**n = 527**	**n = 4,398**	**n = 6,411**	**n = 6,991**
**Diphtheria**	Complete	5,901 (88.5%)	6,129 (89.2%)	4,549 (94.2%)	500 (94.9%)	4,146 (94.3%)	6,064 (94.6%)	6,597 (94.4%)
	Incomplete	332 (5.0%)	342 (5.0%)	76 (1.6%)	7 (1.3%)	59 (1.3%)	87 (1.4%)	112 (1.6%)
	Unvaccinated	436 (6.5%)	397 (5.8%)	202 (4.2%)	20 (3.8%)	193 (4.4%)	260 (4.1%)	282 (4.0%)
**Hepatitis B**	Complete	5,251 (78.7%)	5,502 (80.1%)	4,385 (90.8%)	484 (91.8%)	4,053 (92.2%)	5,903 (92.1%)	6,433 (92.0%)
	Incomplete	752 (11.3%)	769 (11.2%)	111 (2.3%)	12 (2.3%)	82 (1.9%)	132 (2.1%)	150 (2.1%)
	Unvaccinated	666 (10.0%)	597 (8.7%)	331 (6.9%)	31 (5.9%)	263 (6.0%)	376 (5.9%)	408 (5.8%)
**Hib**	Complete	5,698 (85.4%)	5,915 (86.1%)	4,478 (92.8%)	487 (92.4%)	4,076 (92.7%)	5,964 (93.0%)	6,516 (93.2%)
	Incomplete	399 (6.0%)	422 (6.1%)	70 (1.5%)	11 (2.1%)	76 (1.7%)	111 (1.7%)	118 (1.7%)
	Unvaccinated	572 (8.6%)	531 (7.7%)	279 (5.8%)	29 (5.5%)	246 (5.6%)	336 (5.2%)	357 (5.1%)
**MMR**	Complete	5,900 (88.5%)	6,147 (89.5%)	4,439 (92.0%)	494 (93.7%)	4,152 (94.4%)	6,110 (95.3%)	6,683 (95.6%)
	Incomplete	285 (4.3%)	263 (3.8%)	151 (3.1%)	14 (2.7%)	102 (2.3%)	129 (2.0%)	122 (1.7%)
	Unvaccinated	484 (7.3%)	458 (6.7%)	237 (4.9%)	19 (3.6%)	144 (3.3%)	172 (2.7%)	186 (2.7%)
**Meningococcal C**	Complete	5,762 (86.4%)	6,003 (87.4%)	4,300 (89.1%)	481 (91.3%)	3,958 (90.0%)	5,783 (90.2%)	6,310 (90.3%)
	Incomplete	*NA*	*NA*	*NA*	*NA*	*NA*	*NA*	*NA*
	Unvaccinated	907 (13.6%)	865 (12.6%)	527 (10.9%)	46 (8.7%)	440 (10.0%)	628 (9.8%)	681 (9.7%)
**Pertussis**	Complete	5,893 (88.4%)	6,125 (89.2%)	4,542 (94.1%)	500 (94.9%)	4,142 (94.2%)	6,059 (94.5%)	6,589 (94.2%)
	Incomplete	316 (4.7%)	330 (4.8%)	76 (1.6%)	7 (1.3%)	59 (1.3%)	86 (1.3%)	109 (1.6%)
	Unvaccinated	460 (6.9%)	413 (6.0%)	209 (4.3%)	20 (3.8%)	197 (4.5%)	266 (4.1%)	293 (4.2%)
**Pneumococcus**	Complete	5,428 (81.4%)	5,706 (83.1%)	4,104 (85.0%)	447 (84.8%)	3,491 (79.4%)	5,144 (80.2%)	5,616 (80.3%)
	Incomplete	239 (3.6%)	203 (3.0%)	154 (3.2%)	17 (3.2%)	441 (10.0%)	698 (10.9%)	762 (10.9%)

		Year of examination						
**Vaccine**	**Status**	**2018**	**2019**	**2020***	**2021***	**2022***	**2023**	**2024**
		**n = 6,669**	**n = 6,868**	**n = 4,827**	**n = 527**	**n = 4,398**	**n = 6,411**	**n = 6,991**
	Unvaccinated	1,002 (15.0%)	959 (14.0%)	569 (11.8%)	63 (12.0%)	466 (10.6%)	569 (8.9%)	613 (8.8%)
**Polio**	Complete	5,790 (86.8%)	6,014 (87.6%)	4,531 (93.9%)	497 (94.3%)	4,132 (94.0%)	6,030 (94.1%)	6,574 (94.0%)
	Incomplete	410 (6.1%)	415 (6.0%)	74 (1.5%)	7 (1.3%)	60 (1.4%)	96 (1.5%)	110 (1.6%)
	Unvaccinated	469 (7.0%)	439 (6.4%)	222 (4.6%)	23 (4.4%)	206 (4.7%)	285 (4.4%)	307 (4.4%)
**Tetanus**	Complete	5,895 (88.4%)	6,133 (89.3%)	4,551 (94.3%)	499 (94.7%)	4,149 (94.3%)	6,066 (94.6%)	6,596 (94.3%)
	Incomplete	345 (5.2%)	338 (4.9%)	76 (1.6%)	8 (1.5%)	58 (1.3%)	86 (1.3%)	113 (1.6%)
	Unvaccinated	429 (6.4%)	397 (5.8%)	200 (4.1%)	20 (3.8%)	191 (4.3%)	259 (4.0%)	282 (4.0%)
**TBE**	Complete	610 (47.03%)	749 (55.36%)	599 (60.94%)	52 (64.20%)	698 (67.44%)	1,214 (71.45%)	1,341 (68.87%)
	Incomplete	82 (6.32%)	71 (5.25%)	50 (5.09%)	3 (3.70%)	61 (5.89%)	84 (4.94%)	112 (5.75%)
	Unvaccinated	605 (46.65%)	533 (39.39%)	334 (33.98%)	26 (32.10%)	276 (26.67%)	401 (23.60%)	494 (25.37%)
**HAV**	Complete	453 (7.19%)	446 (6.82%)	307 (6.54%)	19 (3.71%)	238 (5.57%)	390 (6.22%)	315 (4.59%)
	Incomplete	239 (3.79%)	216 (3.30%)	191 (4.07%)	12 (2.34%)	134 (3.14%)	223 (3.56%)	240 (3.50%)
	Unvaccinated	5,607 (89.02%)	5,875 (89.87%)	4,195 (89.39%)	481 (93.95%)	3,899 (91.29%)	5,658 (90.23%)	6,311 (91.92%)
**Rota**	Complete	965 (88.37%)	1,255 (89.20%)	1,319 (91.98%)	191 (89.25%)	2,197 (88.48%)	3,621 (91.14%)	4,227 (90.73%)
	Incomplete	81 (7.42%)	107 (7.60%)	86 (6.00%)	18 (8.41%)	206 (8.30%)	237 (5.96%)	291 (6.25%)
	Unvaccinated	46 (4.21%)	45 (3.20%)	29 (2.02%)	5 (2.34%)	80 (3.22%)	115 (2.89%)	141 (3.03%)
**Varicella**	Complete	5,274 (79.1%)	5,610 (81.7%)	4,032 (83.5%)	453 (86.0%)	3,779 (85.9%)	5,575 (87.0%)	6,096 (87.2%)
	Incomplete	409 (6.1%)	353 (5.1%)	290 (6.0%)	33 (6.3%)	240 (5.5%)	300 (4.7%)	294 (4.2%)
	Unvaccinated	986 (14.8%)	905 (13.2%)	505 (10.5%)	41 (7.8%)	379 (8.6%)	536 (8.4%)	601 (8.6%)

Note. Values are n (%) within the given examination year and vaccine unless otherwise indicated. "Complete" denotes the full recommended dose series for the corresponding antigen at the time of examination; "incomplete" denotes partial series completion; "unvaccinated" denotes no recorded dose. NA = not applicable, as a partial (incomplete) category is not defined for meningococcal C vaccination in this dataset. MMR = measles-mumps-rubella combination vaccine; Hib = Haemophilus influenzae type b; HAV= hepatitis A; Rota=rotavirus; TBE= tick-borne encephalitis. * Sample sizes for 2020-2022 were reduced relative to other

years, reflecting disruptions to routine examinations during the COVID-19 pandemic period. Examinations prior to school entry are typically performed within the year of school enrollment.

Table S5. Multinomial logistic regression analysis of vaccination category, relative to only measles, by child, family, and administrative predictors, including interaction effects, for RNHD.

**Predictor**	**n**	**NoMeasles**		**ScheduledNotRota**		**ScheduledPlus**	
		OR (95% CI)	*p*	*OR (95% CI)*	*p*	*OR (95% CI)*	*p*
Child's birthplace							
Unknown	25730	**6.85 (4.79–9.80)**	**<0.001*****	**2.15 (1.93–2.40)**	**<0.001*****	**0.83 (0.77–0.90)**	**<0.001*****
Not Germany	819	1.31 (0.15–11.65)	0.809	1.14 (0.53–2.46)	0.730	**0.59 (0.35–0.99)**	**0.048***
Language family							
East Europe	1096	0.10 (0.00–297.71)	0.569	**2.77 (1.42–5.44)**	**0.003****	**1.76 (1.06–2.94)**	**0.030***
Other	5850	0.25 (0.03–2.39)	0.227	**3.04 (2.22–4.18)**	**<0.001*****	**2.35 (1.84–3.01)**	**<0.001*****
Multilingual + German	5718	0.98 (0.43–2.22)	0.960	**1.30 (1.04–1.62)**	**0.021***	1.11 (0.95–1.30)	0.180
Unknown	715	0.41 (0.00–130000.00)	0.890	1.75 (0.34–9.06)	0.503	1.90 (0.58–6.22)	0.290
Child's media/screen time use							
None	2691	0.88 (0.58–1.34)	0.566	**0.80 (0.66–0.96)**	**0.019***	**0.87 (0.77–1.00)**	**0.045***

Predictor	n	NoMeasles		ScheduledNotRota		ScheduledPlus	
		OR (95% CI)	*p*	*OR (95% CI)*	*p*	*OR (95% CI)*	*p*
1–2 hours	5341	0.83 (0.61–1.14)	0.255	**1.46 (1.27–1.68)**	**<0.001*******	1.11 (0.98–1.25)	0.097
2–4 hours	440	0.22 (0.02–2.58)	0.228	1.20 (0.68–2.13)	0.533	1.04 (0.65–1.66)	0.879
Over 4 hours	97	3.02 (0.49–18.55)	0.233	2.83 (0.78–10.22)	0.112	1.97 (0.60–6.43)	0.262
Unknown	6096	1.05 (0.82–1.34)	0.700	**0.78 (0.68–0.89)**	**<0.001*******	**0.75 (0.68–0.84)**	**<0.001*******
Nationality							
Other	2217	1.42 (0.47–4.29)	0.539	1.12 (0.59–2.14)	0.720	1.01 (0.60–1.71)	0.961
East Europe	494	1.08 (0.02–55.06)	0.969	0.74 (0.06–9.19)	0.814	0.60 (0.10–3.61)	0.580
Mixed German	3290	**0.14 (0.03–0.69)**	**0.016***	0.75 (0.53–1.06)	0.102	**0.79 (0.62–1.00)**	**0.047***
Middle East	353	0.04 (0.00–3.5E11)	0.843	0.32 (0.00–92.03)	0.695	1.11 (0.09–13.95)	0.933
Unknown	963	1.20 (0.63–2.29)	0.586	0.69 (0.45–1.06)	0.087	0.99 (0.73–1.34)	0.947
Parental employment							
One parent FT or PT	8814	1.24 (0.96–1.61)	0.098	0.94 (0.84–1.06)	0.336	**0.87 (0.79–0.96)**	**0.005****
Both parents 1 FT + 1 PT	17310	0.95 (0.75–1.20)	0.659	0.98 (0.88–1.10)	0.756	1.07 (0.98–1.16)	0.142

Predictor	n	NoMeasles		ScheduledNotRota		ScheduledPlus	
		OR (95% CI)	*p*	*OR (95% CI)*	*p*	*OR (95% CI)*	*p*
Unknown	7536	1.09 (0.75–1.58)	0.654	**0.70 (0.58–0.84)**	**<0.001*** **	**0.66 (0.57–0.77)**	**<0.001*** **
Social status							
Medium	7900	0.90 (0.72–1.11)	0.308	**1.17 (1.07–1.29)**	**0.001** **	1.02 (0.95–1.11)	0.560
Low	3302	0.97 (0.72–1.30)	0.851	**1.47 (1.28–1.69)**	**<0.001*** **	**1.15 (1.03–1.30)**	**0.016* **
Unknown	7991	0.98 (0.70–1.37)	0.892	1.10 (0.94–1.30)	0.234	1.14 (1.00–1.30)	0.052
Medical exam status							
Unknown	847	1.57 (0.83–2.96)	0.161	0.78 (0.51–1.19)	0.243	**0.43 (0.30–0.61)**	**<0.001*** **
Incomplete	8043	1.15 (0.93–1.42)	0.186	**0.75 (0.67–0.84)**	**<0.001*** **	**0.63 (0.58–0.69)**	**<0.001*** **
Significant interaction effects – Language Family:							
East Europe × Birthplace: Not Germany	146	47.08 (0.01–2.0E5)	0.370	0.42 (0.13–1.34)	0.143	**0.08 (0.03–0.21)**	**<0.001*** **
East Europe × Media: 1–2 hours	306	1.03 (0.37–2.86)	0.957	**0.53 (0.32–0.89)**	**0.015* **	**0.64 (0.41–0.99)**	**0.045* **
East Europe × Media: 2–4 hours	45	6.98 (0.35–139.97)	0.204	**0.16 (0.03–0.85)**	**0.031* **	0.57 (0.22–1.48)	0.246

**Predictor**	**n**	**NoMeasles**		**ScheduledNotRota**		**ScheduledPlus**	
		OR (95% CI)	***p***	***OR (95% CI)***	***p***	***OR (95% CI)***	***p***
East Europe × Nationality: Other	84	0.31 (0.05–1.85)	0.200	**0.24 (0.08–0.69)**	**0.008****	**0.40 (0.17–0.90)**	**0.026***
Multilingual+German × Birthplace: Not Germany	188	0.39 (0.02–8.92)	0.560	**0.37 (0.15–0.93)**	**0.035***	**0.21 (0.11–0.40)**	**<0.001*****
Multilingual+German × Media: 1–2 hours	534	0.84 (0.18–3.88)	0.819	**0.55 (0.36–0.82)**	**0.004****	0.95 (0.72–1.26)	0.713
Multilingual+German × Nationality: Mixed German	1841	2.63 (0.44–15.84)	0.292	1.08 (0.71–1.63)	0.732	**1.35 (1.01–1.80)**	**0.039***
Other × Birthplace: Not Germany	389	4.66 (0.19–112.51)	0.344	**0.31 (0.13–0.75)**	**0.009****	**0.14 (0.07–0.26)**	**<0.001*****
Other × Media: 1–2 hours	1457	1.17 (0.69–1.99)	0.568	**0.78 (0.62–1.00)**	**0.049***	0.83 (0.68–1.03)	0.088
Other × Media: None	130	0.69 (0.08–5.96)	0.739	**2.04 (1.05–3.96)**	**0.036***	**2.06 (1.20–3.54)**	**0.008****
Other × Media: Over 4 hours	46	1.18 (0.15–9.60)	0.876	0.31 (0.07–1.45)	0.137	**0.24 (0.06–1.00)**	**0.050***
Other × Medical exam: Incomplete	1764	0.76 (0.49–1.19)	0.234	1.03 (0.84–1.27)	0.789	**0.80 (0.68–0.96)**	**0.013***
Other × Nationality: Mixed German	816	5.14 (0.97–27.22)	0.055	**0.60 (0.39–0.92)**	**0.019***	**0.68 (0.49–0.94)**	**0.018***

Predictor	n	NoMeasles		ScheduledNotRota		ScheduledPlus	
		OR (95% CI)	*p*	*OR (95% CI)*	*p*	*OR (95% CI)*	*p*
Other × Nationality: Other	1471	0.51 (0.16–1.65)	0.260	**0.30 (0.15–0.58)**	**<0.001*****	**0.39 (0.23–0.68)**	**0.001*****

Note. OR = odds ratio; CI = confidence interval; n = number of children in the predictor category (denominator common to all three outcomes). Reference outcome for the multinomial model is full/on-time vaccination per the recommended schedule. Reference categories: Child born in Germany; German family language; <1 hour/day media/screen time; both parents full-time employed; German nationality; high social status; complete medical exam. Bold values indicate statistically significant associations (p < 0.05): * p<0.05, ** p<0.01, *** p<0.00

Table S6. Multinomial logistic regression analysis of vaccination category, relative to only measles, by child, family, and administrative predictors, including interaction effects, for FFM

Predictor	n	NotMeasles		ScheduledNotRota		ScheduledPlus	
		OR (95% CI)	*p*	*OR (95% CI)*	*p*	*OR (95% CI)*	*p*
Intercept							
Intercept	35449	0.09 (0.07–0.11)	<0.001***	0.97 (0.88–1.07)	0.696	**2.37 (2.16–2.60)**	**<0.001*****
Child's country/region of birth							
EastEurope	1461	**2.28 (1.00–5.21)**	**0.051**	1.14 (0.72–1.81)	0.567	1.00 (0.62–1.62)	0.993
AfricaOrAsia	1383	0.90 (0.20–3.97)	0.891	0.95 (0.55–1.63)	0.839	1.03 (0.60–1.76)	0.920

Predictor	n	NotMeasles		ScheduledNotRota		ScheduledPlus	
		OR (95% CI)	*p*	*OR (95% CI)*	*p*	*OR (95% CI)*	*p*
Turkey	97	0.73 (0.00–1116.53)	0.933	1.70 (0.21–13.90)	0.623	1.27 (0.14–11.61)	0.830
Other	237	0.36 (0.00–744.86)	0.795	1.50 (0.16–13.73)	0.721	4.16 (0.51–33.74)	0.182
Arabic	373	0.32 (0.00–31.33)	0.629	0.90 (0.32–2.54)	0.845	0.91 (0.31–2.71)	0.871
Unknown	65	0.13 (0.00–5.80)	0.295	0.72 (0.36–1.47)	0.372	0.45 (0.19–1.05)	0.064
Family language							
Not German	18683	1.17 (0.94–1.44)	0.153	**1.52 (1.38–1.67)**	**<0.001**	1.01 (0.92–1.11)	0.799
Unknown	1162	0.56 (0.39–0.82)	0.002	0.99 (0.83–1.18)	0.886	**1.26 (1.06–1.51)**	**0.010**
Kindergarten attendance							
Unknown	1393	**2.67 (1.77–4.03)**	**<0.001**	0.77 (0.59–0.99)	0.044	**0.72 (0.55–0.94)**	**0.016**
Between 0-2 years	3580	0.86 (0.60–1.24)	0.416	**0.83 (0.71–0.96)**	**0.015**	**0.83 (0.71–0.97)**	**0.020**
Between 2-3 years	7755	0.86 (0.68–1.08)	0.187	0.90 (0.82–1.00)	0.045	0.95 (0.86–1.05)	0.312
Over 4 years	12629	**0.65 (0.53–0.80)**	**<0.001**	**0.68 (0.63–0.75)**	**<0.001**	**1.12 (1.03–1.22)**	**0.010**
Father's nationality							
Other	615	1.36 (0.91–2.05)	0.135	**0.70 (0.55–0.89)**	**0.004**	0.90 (0.72–1.12)	0.356
EastEurope	5182	0.97 (0.77–1.22)	0.798	**1.43 (1.29–1.60)**	**<0.001**	1.08 (0.97–1.21)	0.149
Unknown	1085	0.97 (0.69–1.37)	0.850	**1.48 (1.24–1.77)**	**<0.001**	1.05 (0.88–1.26)	0.608

**Predictor**	**n**	**NotMeasles**		**ScheduledNotRota**		**ScheduledPlus**	
		OR (95% CI)	*p*	*OR (95% CI)*	*p*	*OR (95% CI)*	*p*
Arabic	3096	0.80 (0.60–1.05)	0.108	**1.40 (1.23–1.58)**	**<0.001**	**1.14 (1.01–1.29)**	**0.037**
AfricaOrAsia	5600	**0.77 (0.61–0.97)**	**0.026**	**1.37 (1.23–1.52)**	**<0.001**	**1.37 (1.23–1.52)**	**<0.001**
Turkey	2275	0.68 (0.46–1.00)	0.051	**2.00 (1.73–2.31)**	**<0.001**	**1.48 (1.28–1.71)**	**<0.001**
Number of siblings							
TwoOrMoreSiblings	11672	1.05 (0.91–1.20)	0.525	**1.14 (1.06–1.22)**	**<0.001**	**0.92 (0.86–0.99)**	**0.028**
OnlyChild	6324	1.01 (0.87–1.19)	0.865	1.02 (0.94–1.12)	0.571	0.98 (0.91–1.07)	0.664
Unknown	316	0.73 (0.40–1.33)	0.302	1.35 (0.98–1.84)	0.065	0.79 (0.56–1.12)	0.182
Caregiver's German language ability							
Unknown	18315	**2.01 (1.64–2.47)**	**<0.001**	**2.71 (2.47–2.98)**	**<0.001**	**0.86 (0.78–0.94)**	**0.002**
Basic	1383	1.02 (0.75–1.37)	0.920	1.09 (0.89–1.35)	0.405	1.18 (0.97–1.43)	0.099
Moderate	4765	0.85 (0.65–1.11)	0.222	**1.20 (1.06–1.35)**	**0.003**	**1.41 (1.26–1.57)**	**<0.001**
Child's German language ability							
Unknown	10191	**1.49 (1.20–1.85)**	**<0.001**	0.99 (0.89–1.10)	0.871	**0.76 (0.68–0.84)**	**<0.001**
Low	3243	**1.29 (1.07–1.55)**	**0.009**	**0.82 (0.72–0.93)**	**0.003**	**0.80 (0.70–0.91)**	**<0.001**
Medical exam status							
Unknown	2065	1.20 (0.74–1.94)	0.457	**0.31 (0.23–0.43)**	**<0.001**	**0.43 (0.31–0.59)**	<0.001
Incomplete	5716	1.26 (0.95–1.68)	0.111	**0.59 (0.50–0.69)**	**<0.001**	**0.45 (0.38–0.53)**	<0.001
Interaction effects (medical exam status × other predictors)							
Medical exam: Incomplete × Birthplace: Unknown	11	17.5 (0.28–1.1E3)	0.177	0.83 (0.17–4.03)	0.818	0.61 (0.06–6.79)	0.689

Predictor	n	NotMeasles		ScheduledNotRota		ScheduledPlus	
		OR (95% CI)	*p*	*OR (95% CI)*	*p*	*OR (95% CI)*	*p*
Medical exam: Incomplete × Birthplace: Turkey	51	1.8 (0.00–2.9E10)	0.878	**0.10 (0.01–0.94)**	**0.044**	0.12 (0.01–1.25)	0.075
Medical exam: Incomplete × Birthplace: AfricaOrAsia	746	0.93 (0.21–4.20)	0.929	**0.09 (0.05–0.16)**	**<0.001**	**0.16 (0.09–0.29)**	**<0.001**
Medical exam: Incomplete × Birthplace: Arabic	204	2.69 (0.03–266.28)	0.673	**0.09 (0.03–0.27)**	**<0.001**	**0.14 (0.04–0.47)**	**0.001**
Medical exam: Incomplete × Birthplace: EastEurope	826	0.46 (0.20–1.09)	0.077	**0.07 (0.04–0.11)**	**<0.001**	**0.06 (0.03–0.11)**	**<0.001**
Medical exam: Incomplete × Birthplace: Other	159	1.56 (0.0–3.3E10)	0.910	**0.06 (0.01–0.63)**	**0.018**	**0.06 (0.01–0.49)**	**0.009**
Medical exam: Incomplete × Kindergarten: Over 4 years	1086	1.15 (0.74–1.79)	0.527	**1.32 (1.05–1.66)**	**0.016**	1.24 (0.99–1.55)	0.067
Medical exam: Incomplete × Kindergarten: Between 2-3 years	1549	0.95 (0.65–1.39)	0.801	0.82 (0.66–1.02)	0.070	0.83 (0.66–1.04)	0.109
Medical exam: Incomplete × Kindergarten: Between 0-2 years	1189	1.18 (0.74–1.88)	0.488	**0.57 (0.43–0.74)**	**<0.001**	**0.70 (0.53–0.93)**	**0.013**

Note. OR = odds ratio; CI = confidence interval; n = number of children in the predictor category (denominator common to all three outcomes). Reference outcome for the multinomial model is full/on-time vaccination per the recommended schedule. Reference categories: German family language; complete medical exam; German father's nationality; child born in Germany; good caregiver/child German language ability. Bold values indicate statistically significant associations ($p < 0.05$): * $p<0.05$, ** $p<0.01$, *** $p<0.001$.
